# Household and Community Clustering of Concurrent Vaccine Hesitancy in China: Multicenter Survey

**DOI:** 10.2196/104263

**Published:** 2026-10-07

**Authors:** Yijing Zhang, Soumitra S Bhuyan, Saiyi Wang, Xinghong Guo, Tingting Cao, Dongfang Zhu, Yudong Miao, Jianping Hu, Wei Wang, Quanman Li, Jian Wu

**Affiliations:** 1 Department of Health Policy and Management, College of Public Health, Zhengzhou University, No. 100 Science Avenue, Gaoxin District, Zhengzhou, Henan, 450001, China, 86 67739815; 2 School of Public and International Affairs, Princeton University, Princeton, NJ, United States; 3 Edward J. Bloustein School of Planning and Public Policy, Rutgers, The State University of New Jersey, New Brunswick, NJ, United States; 4 Henan Medical Communication and Project Forward Center, Zhengzhou, Henan, China

**Keywords:** vaccine hesitancy, community clustering, household clustering, multilevel analysis, network analysis

## Abstract

**Background:**

Vaccine hesitancy remains a persistent global challenge, especially in the context of major public health emergencies. While individual-level factors have been widely studied, whether and how household and community contexts shape hesitancy clustering has received little attention.

**Objective:**

This study aimed to assess household- and community-level clustering of COVID-19 booster vaccine hesitancy (CBVH), identify key associated factors, and explore potential intervention targets.

**Methods:**

In March 2023, a household-based cross-sectional survey was conducted among Chinese adults across 4 provinces. Concurrent CBVH was defined as hesitancy reported for both current and future booster intentions within the same survey. The analytical samples included 5462 participants for current CBVH, 5437 for future CBVH, and 5407 for concurrent CBVH. Three-level logistic regression models were used to quantify variance attributable to individual, household, and community levels and to identify associated factors. Network analysis was also used to examine interrelationships among key factors and identify central intervention targets.

**Results:**

Among 5407 participants, 1461 (27.0%) exhibited concurrent CBVH. The unweighted prevalence of current and future CBVH was 28.4% (1553/5462) and 30.1% (1636/5437), respectively. The corresponding poststratified prevalence estimates were 30.88% and 32.55%, respectively. In the null model, the combined household- and community-level intraclass correlation coefficients were 77.73% (95% CI 72.40%‐82.29%) and 78.84% (95% CI 73.57%‐83.30%), respectively. Prior COVID-19 vaccination, self-efficacy, perceived benefits, trust in vaccine developers, and knowledge of the vaccine were associated with lower CBVH, while perceived barriers, perceived severity, household income, chronic disease, history of allergies, and living in the eastern region were associated with higher hesitancy. Network analysis revealed that self-efficacy and prior COVID-19 vaccination had the strongest associations with CBVH. Perceived barriers and trust in vaccine developers formed the strongest association among nodes. The current and future CBVH networks showed similar configurations.

**Conclusions:**

The substantial household and community clustering highlights the need for group interventions. Household-centered vaccine education, community-based vaccine counseling services, and transparent vaccine communication may help reduce vaccine hesitancy and strengthen preparedness for future infectious disease outbreaks.

## Introduction

Vaccine hesitancy, defined as the delay in acceptance or refusal of vaccination despite the availability of immunization services [1], was recognized by the World Health Organization as one of the top 10 threats to global health [2]. Critically, its principal risk is driving immunization coverage below the herd immunity threshold, ultimately compromising population-level protection [3]. The COVID-19 pandemic brought this issue into sharp focus. While vaccines were estimated to have prevented millions of deaths, vaccine hesitancy persisted and has continued into the postpandemic era. Recent evidence suggests that vaccine hesitancy may persist over time and extend beyond a single pathogen or vaccination context [4]. A cohort study of over 1.1 million individuals in England found that concerns about vaccine effectiveness and health effects declined over time, whereas hesitancy linked to low trust, low perceived risk, and broader opposition to vaccination was more persistent [5]. A multinational survey spanning 23 countries with 23,000 participants revealed that among individuals who had completed their primary vaccination series, the COVID-19 booster vaccine hesitancy (CBVH) rate was 12.1%, with Russia exhibiting the highest rate at 28.9% [6]. In China, a repeated cross-sectional study demonstrated an increase in COVID-19 vaccine hesitancy, rising from 8.39% in 2021 to 29.72% in 2023 [7]. Therefore, understanding vaccine hesitancy is critical not only for addressing remaining COVID-19 challenges but also for strengthening global preparedness against future outbreaks.

Existing studies have identified the individual correlates of COVID-19 vaccine hesitancy [7-9], including sociodemographic characteristics (eg, sex, age, and education level), health-related factors (eg, chronic diseases, drinking, and smoking), trust in the medical system, and exposure to misinformation. In addition, the Health Belief Model posits that psychological perceptions, including perceived susceptibility, perceived severity, perceived benefits, perceived barriers, cues to action, and self-efficacy, are key factors in vaccine hesitancy. The Social Ecological Model further recognizes that health behaviors are shaped not only by individual factors but also by interpersonal relationships and community contexts [10]. At the household level, shared information, norms, health experiences, and decision-making processes may produce similar attitudes toward vaccination among family members [11]. However, most household-level research has focused on parental decisions about childhood vaccination or has treated family influence as an individual-level exposure [12,13]. At the community level, community engagement may promote vaccine uptake and ensure the effective implementation of health equity interventions [14]. A study involving 7241 residents from 71 communities in China found that community-level differences accounted for 2.4% to 3.7% of the variation in COVID-19 vaccine refusal and 8.5% to 9.6% of the variation in delayed COVID-19 vaccination [15]. Other studies have reported geographic clustering and associations with regional socioeconomic conditions, racial or ethnic composition, political orientation, and religious context [16,17]. Few studies, however, have examined individual-, household-, and community-level clustering within a multilevel framework. A study conducted in the Dominican Republic investigated COVID-19 vaccine hesitancy using a 3-level multilevel framework and demonstrated significant spatial clustering [18]. However, the relative contributions of household and community contexts to overall clustering were not comprehensively characterized. More importantly, no study has evaluated these multilevel interactions within China, a unique sociocultural and administrative landscape characterized by a strong collectivist culture, family-oriented decision-making, and community-based health governance [19]. Crucially, it remains unclear to what extent CBVH clusters within households and communities and how its associated individual-, household-, and community-level factors are conditionally interconnected. Addressing these gaps may provide important evidence for designing tailored, multitiered immunization strategies and optimizing precision public health interventions in China and similar settings.

In this study, we analyzed data from a multicenter, household-based survey conducted across 4 provinces in China. This study aimed to assess the extent of household- and community-level clustering of CBVH, identify key factors associated with CBVH, and further explore interrelationship patterns and identify central nodes as potential intervention targets. By integrating multilevel and network approaches, we sought to provide a more comprehensive understanding of the factors associated with vaccine hesitancy and to inform multilevel intervention planning for future pandemic responses and vaccination campaigns.

## Methods

### Procedures and Participants

We used a stratified random sampling method to conduct a multicenter, observational study of vaccine hesitancy in Chinese households. The study was reported in accordance with the STROBE (Strengthening the Reporting of Observational Studies in Epidemiology) guidelines (Checklist 1). Four cities were selected from China’s 4 major economic regions: Eastern (Changzhou, Jiangsu), Central (Zhengzhou, Henan), Western (Xining, Qinghai), and Northeastern (Mudanjiang, Heilongjiang). These regions differ in economic development, health care resources, and cultural norms. The minimum sample sizes for each region were determined based on the population proportions of China’s Seventh National Population Census (2020). Within each city, at least 2 urban and 2 rural areas were randomly selected for sampling, followed by a random selection of households within each identified area. All eligible members aged 18 years and above were invited to participate, as vaccination decisions for minors are generally made by parents or guardians [20]. This survey was conducted in March 2023. Participants completed either an online questionnaire administered through the Wenjuanxing platform or a paper questionnaire with the assistance of survey staff. To reduce selection and information bias, trained staff thoroughly reviewed all questionnaires based on the exclusion criteria. Finally, a total of 5891 participants aged 18 years and above from 2387 households were enrolled.

Following rigorous data cleaning to exclude invalid questionnaires, 5780 participants were included in this analysis. We further excluded participants with missing data on current and future CBVH, yielding a final analytical sample of 5462 participants for the analysis of current CBVH and 5437 participants for the analysis of future CBVH. Among them, 5407 participants responded to both questions and were subsequently included in the concurrent CBVH analysis. The detailed participant flow is shown in Figure 1.

**Figure 1.**
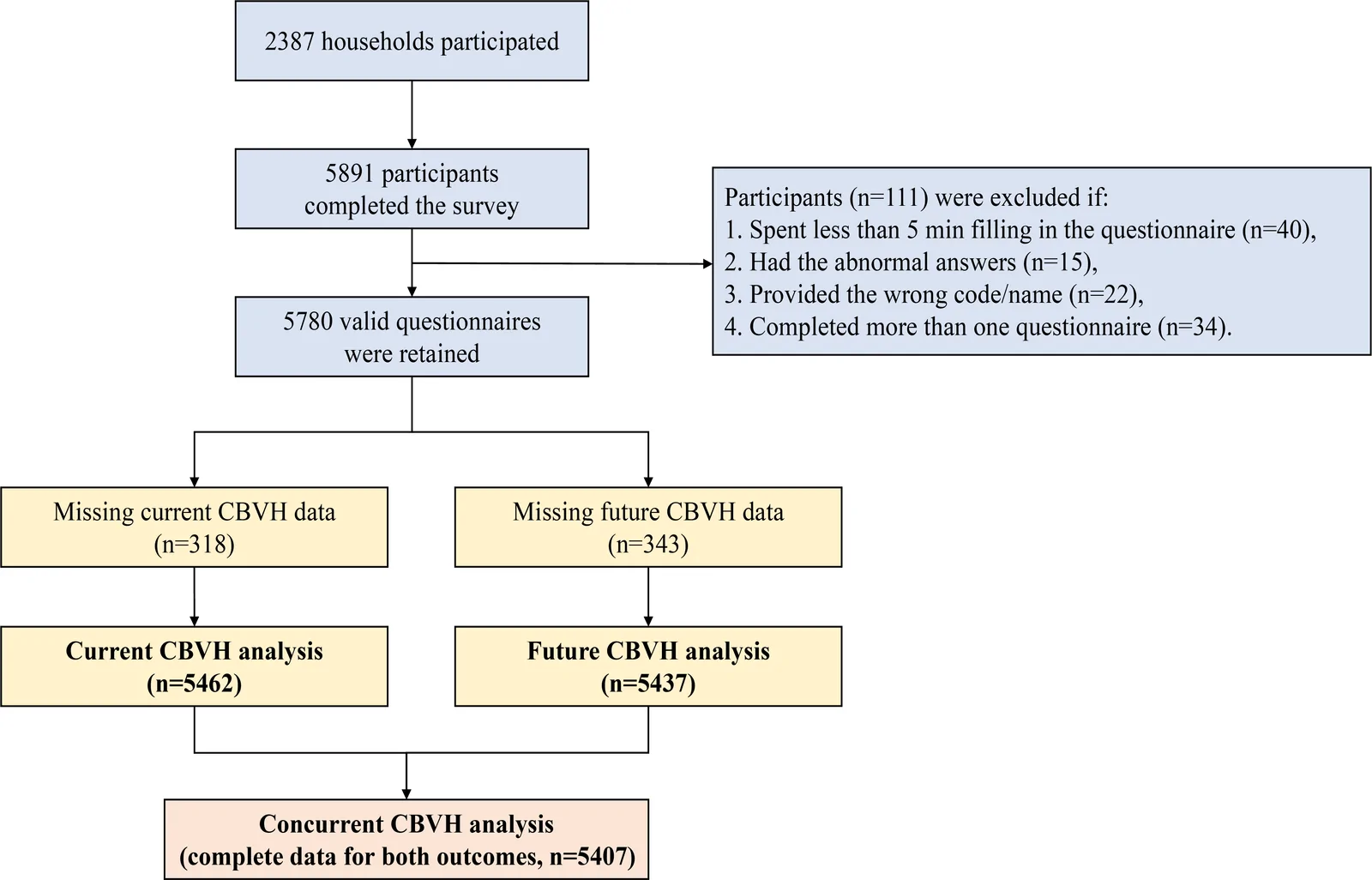
Flowchart of participant selection. CBVH: COVID-19 booster vaccine hesitancy.

### Ethical Considerations

This study was conducted in accordance with the Declaration of Helsinki. This survey project was approved by the Life Science Ethics Review Committee of Zhengzhou University (approval number: 2021-01-12-05), and all participants provided written informed consent. All data were handled confidentially, and no compensation was provided.

### Assessments

#### Outcomes

The primary outcomes were current and future CBVH, which were self-reported. Participants were asked 2 questions: “At present, would you like to get a booster vaccination of the COVID-19 vaccine?” and “In the future, would you like to get a booster vaccination of the COVID-19 vaccine?” Participants could choose one response from the following options: “(1) willing,” “(2) hesitant or delayed,” “(3) refused,” and “(4) not applicable.” According to the definition of vaccine hesitancy [9], option (1) was regarded as “acceptance,” and options (2) and (3) were merged into “hesitancy.” In addition, participants were excluded if they selected the option “(4) not applicable.” In this study, concurrent CBVH was defined as hesitancy reported for both current and future booster intentions within the same survey.

#### Covariates

The questionnaire included (1) sociodemographic characteristics: age, sex, ethnicity, religion, marital status, educational level, employment status, region, coresidence status, number of children, number of older adults, annual household income, smoking status, and drinking status; (2) health conditions: history of allergies, chronic diseases, and prior COVID-19 vaccination status; (3) awareness of the COVID-19 vaccine: sources of COVID-19 vaccine information and knowledge of the COVID-19 vaccine; (4) self-perception: perceived severity, perceived susceptibility, perceived benefits, perceived barriers, self-efficacy, and cues to action; (5) trust in the medical system: trust in doctors and vaccine developers; and (6) convenience of vaccination: distance and time to the vaccination point. Details of the items, response formats, scoring, and internal consistency are provided in Table S1 in Multimedia Appendix 1. Vaccine knowledge and self-perception variables were categorized using quartile-based cutoffs.

### Statistical Analysis

Descriptive analyses were conducted to characterize the study sample. For continuous variables, means and SDs were calculated; for categorical variables, frequencies and percentages were reported. Chi-square and 2-tailed independent-samples *t* tests were used to examine differences between participants with and without CBVH. Poststratification weighting was used to adjust the sample according to the age, sex, and region distributions reported in the Seventh National Population Census of China (2020) [21]. Participants with missing outcome data were excluded from the corresponding outcome-specific analysis. No covariate data were missing after data cleaning.

A 3-level logistic regression modeling approach was used due to the hierarchical structure of our data. Specifically, individuals were nested within households, and households within communities. Fixed effects were presented as adjusted odds ratios (aORs) with corresponding 95% CIs. Random effects were assessed using random-intercept variances, the variance partition coefficient (VPC), the intraclass correlation coefficient (ICC), the median odds ratio (MOR), and proportional changes in variance (PCV). The community- and household-level VPCs indicate the proportions of overall variation attributable to differences between communities and households, respectively. The combined ICC represents clustering among individuals in the same household and includes both household- and community-level variances [22]. The MOR represents the median odds ratio between higher- and lower-risk clusters and quantifies unexplained heterogeneity on the odds-ratio scale, whereas the PCV quantifies the proportional change in the corresponding variance relative to model 0 [23]. The individual-level residual variance was fixed at π^2^/3, and 95% CIs were estimated using the delta method. Four models were fitted sequentially. First, a null model with only household- and community-level random intercepts was fitted to partition the variance. Subsequently, 3 multivariable models were built: model 1 adjusted for individual-level factors, including age, sex, ethnicity, religion, marital status, educational level, employment status, coresidence status, smoking status, drinking status, chronic disease, history of allergies, prior COVID-19 vaccination status, source of COVID-19 vaccine information, knowledge of COVID-19 vaccine, perceived severity, perceived susceptibility, perceived benefits, perceived barriers, self-efficacy, cues to action, trust in doctors, and trust in vaccine developers; model 2 further adjusted for household-level factors, including number of children, number of older adults, and annual household income; and model 3 further adjusted for community-level factors, including region, distance to vaccination point, and time to vaccination point. Model fit was evaluated using the Akaike information criterion (AIC), Bayesian information criterion (BIC), and log-likelihood (LL), with better fit indicated by lower AIC and BIC values and higher log-likelihood.

Then, network analysis was conducted to visualize and quantify complex interrelationships among factors that were statistically significantly associated with CBVH within multilevel models. The current and future CBVH networks were estimated using mixed graphical models, which allow continuous, binary, and multicategorical variables within a single analytical framework [24]. Only pairwise interactions were estimated (*k*=2). Least absolute shrinkage and selection operator regularization [25], combined with extended Bayesian information criterion model selection (γ=0.50), were applied to obtain conservative and sparse network estimates [26]. Nodes represented the included variables, whereas edges represented pairwise conditional associations after controlling for all other variables in the network. Positive and negative associations were represented by green and red edges, respectively, with thicker edges indicating stronger associations. For multicategorical variables, the edge weight was summarized using the mean absolute magnitude of category-specific parameters and was displayed in gray because a single direction could not be assigned. Node predictability indicates the extent to which a node could be predicted by its adjacent nodes [27]. It was quantified using the proportion of explained variance (*R*^2^) for continuous variables and the proportion of correct classifications (CC) for categorical variables. Centrality indices, including strength, betweenness, and closeness, were calculated to identify the most influential nodes within the network. Edge-weight accuracy was evaluated using nonparametric bootstrapping with 1000 replicates, from which 95% CIs were obtained. The correlation stability coefficient (CS-C) was reported, with a threshold of 0.25 or higher, and ideally above 0.50 [28]. We also ranked the edges directly connected to the CBVH node and displayed them in a heatmap [29]. Signs were retained for continuous and binary variables, while multicategorical edges were summarized using mean absolute parameter values and compared descriptively.

To evaluate the robustness of the primary findings, 3 sensitivity analyses were performed. Because religious beliefs [30] and allergy history [31] have been identified as important determinants of vaccine hesitancy, the analyses were repeated after separately excluding participants reporting religious beliefs and those with a history of allergies. Participants from the eastern region were excluded to assess the robustness of the results with respect to the uneven regional sample distribution. All statistical analyses were performed using Stata (version 18.0; StataCorp LLC) and R (version 4.4.2; R Foundation for Statistical Computing), with a 2-tailed *P* value of <.05 considered statistically significant.

## Results

### Characteristics of Participants

The unweighted prevalence of current and future CBVH was 28.4% (1553/5462) and 30.1% (1636/5437), respectively. After poststratification, the corresponding prevalence estimates were 30.88% and 32.55%, respectively (Figure S1 in Multimedia Appendix 1). Among the 5407 participants with complete data for both outcomes, 1461 (27.0%) exhibited concurrent CBVH. In the current CBVH analytical sample (n=5462), participants were distributed across all age groups, and 46.5% (n=2538) were male participants. Most participants were married (n=4832, 88.5%) and employed (n=5038, 92.2%). The future CBVH analytical sample (n=5437) showed a similar distribution. For both groups, we found that age, educational level, employment, region, number of children, annual household income, smoking status, drinking status, history of allergies, chronic disease, prior COVID-19 vaccination status, source of COVID-19 vaccine information, knowledge of the COVID-19 vaccine, perceived severity, perceived susceptibility, perceived benefits, perceived barriers, self-efficacy, cues to action, trust in doctors, trust in vaccine developers, distance to the vaccination point, and time to the vaccination point all differed between participants with and without CBVH (all *P*<.05). The detailed characteristics are presented in Table 1.

**Table 1.** Characteristics of participants by current and future COVID-19 booster vaccine hesitancy (CBVH) status^a^.

Covariates	Current CBVH				Future CBVH			
	Total, n (%)	No, n (%)	Yes, n (%)	*P* value	Total, n (%)	No, n (%)	Yes, n (%)	*P* value
All participants	5462 (100)	3909 (71.6)	1553 (28.4)	—^b^	5437 (100)	3801 (69.9)	1636 (30.1)	—
Sociodemographic characteristics								
Age (y)				<.001				.002
18‐29	678 (12.4)	509 (13.0)	169 (10.9)		680 (12.5)	498 (13.1)	182 (11.1)	
30‐39	1475 (27.0)	1013 (25.9)	462 (29.7)		1470 (27.0)	983 (25.9)	487 (29.8)	
40‐49	1060 (19.4)	775 (19.8)	285 (18.4)		1056 (19.4)	741 (19.5)	315 (19.3)	
50‐59	1162 (21.3)	864 (22.1)	298 (19.2)		1157 (21.3)	845 (22.2)	312 (19.1)	
≥60	1087 (19.9)	748 (19.1)	339 (21.8)		1074 (19.8)	734 (19.3)	340 (20.8)	
Sex				.09				.12
Male	2538 (46.5)	1845 (47.2)	693 (44.6)		2525 (46.4)	1792 (47.1)	733 (44.8)	
Female	2924 (53.5)	2064 (52.8)	860 (55.4)		2912 (53.6)	2009 (52.9)	903 (55.2)	
Ethnicity				.05				.09
Han	5314 (97.3)	3792 (97.0)	1522 (98.0)		5292 (97.3)	3690 (97.1)	1602 (97.9)	
Minority	148 (2.7)	117 (3.0)	31 (2.0)		145 (2.7)	111 (2.9)	34 (2.1)	
Religion				.01				.09
Nonreligious	5212 (95.4)	3712 (95.0)	1500 (96.6)		5189 (95.4)	3615 (95.1)	1574 (96.2)	
Others	250 (4.6)	197 (5.0)	53 (3.4)		248 (4.6)	186 (4.9)	62 (3.8)	
Marital status				.14				.12
Married	4832 (88.5)	3442 (88.1)	1390 (89.5)		4814 (88.5)	3348 (88.1)	1466 (89.6)	
Others	630 (11.5)	467 (11.9)	163 (10.5)		623 (11.5)	453 (11.9)	170 (10.4)	
Educational level				.002				<.001
Below high school	2371 (43.4)	1750 (44.8)	621 (40.0)		2347 (43.2)	1714 (45.1)	633 (38.7)	
High school	1381 (25.3)	983 (25.1)	398 (25.6)		1382 (25.4)	958 (25.2)	424 (25.9)	
University and above	1710 (31.3)	1176 (30.1)	534 (34.4)		1708 (31.4)	1129 (29.7)	579 (35.4)	
Employment				<.001				<.001
Unemployed	424 (7.8)	244 (6.2)	180 (11.6)		424 (7.8)	237 (6.2)	187 (11.4)	
Employed	5038 (92.2)	3665 (93.8)	1373 (88.4)		5013 (92.2)	3564 (93.8)	1449 (88.6)	
Region				<.001				<.001
Eastern	2072 (37.9)	1248 (31.9)	824 (53.1)		2061 (37.9)	1200 (31.6)	861 (52.6)	
Central	2335 (42.7)	1924 (49.2)	411 (26.5)		2323 (42.7)	1878 (49.4)	445 (27.2)	
Western	168 (3.1)	120 (3.1)	48 (3.1)		165 (3.0)	117 (3.1)	48 (2.9)	
Northeastern	887 (16.2)	617 (15.8)	270 (17.4)		888 (16.3)	606 (15.9)	282 (17.2)	
Coresidence				.93				.51
With household members	5371 (98.3)	3843 (98.3)	1528 (98.4)		5346 (98.3)	3734 (98.2)	1612 (98.5)	
Alone	91 (1.7)	66 (1.7)	25 (1.6)		91 (1.7)	67 (1.8)	24 (1.5)	
Number of children	1.0 (1.1)	1.0 (1.1)	0.8 (1.0)	<.001	1.0 (1.1)	1.0 (1.1)	0.9 (1.0)	<.001
Number of older adults	0.7 (0.9)	0.7 (0.9)	0.6 (0.9)	.47	0.7 (0.9)	0.7 (0.9)	0.6 (0.9)	.58
Annual household income (10,000 CNY^c^)	11.8 (25.5)	10.5 (21.1)	15.3 (34.0)	<.001	11.8 (25.6)	10.4 (21.2)	15.2 (33.4)	<.001
Smoking status				.01				.03
Never smoker	4048 (74.1)	2909 (74.4)	1139 (73.3)		4025 (74.0)	2834 (74.6)	1191 (72.8)	
Former smoker	310 (5.7)	199 (5.1)	111 (7.1)		310 (5.7)	196 (5.2)	114 (7.0)	
Current smoker	1104 (20.2)	801 (20.5)	303 (19.5)		1102 (20.3)	771 (20.3)	331 (20.2)	
Drinking status				.02				.01
Never drinker	3795 (69.5)	2728 (69.8)	1067 (68.7)		3774 (69.4)	2662 (70.0)	1112 (68.0)	
Former drinker	285 (5.2)	183 (4.7)	102 (6.6)		286 (5.3)	178 (4.7)	108 (6.6)	
Current drinker	1382 (25.3)	998 (25.5)	384 (24.7)		1377 (25.3)	961 (25.3)	416 (25.4)	
Health conditions								
History of allergies				<.001				<.001
No	5093 (93.2)	3703 (94.7)	1390 (89.5)		5067 (93.2)	3603 (94.8)	1464 (89.5)	
Yes	369 (6.8)	206 (5.3)	163 (10.5)		370 (6.8)	198 (5.2)	172 (10.5)	
Chronic disease				<.001				<.001
No	4527 (82.9)	3331 (85.2)	1196 (77.0)		4511 (83.0)	3239 (85.2)	1272 (77.8)	
Yes	935 (17.1)	578 (14.8)	357 (23.0)		926 (17.0)	562 (14.8)	364 (22.2)	
Prior COVID-19 vaccination status				<.001				<.001
No	185 (3.4)	83 (2.1)	102 (6.6)		182 (3.3)	81 (2.1)	101 (6.2)	
Yes	5277 (96.6)	3826 (97.9)	1451 (93.4)		5255 (96.7)	3720 (97.9)	1535 (93.8)	
Awareness of COVID-19 vaccine								
Source of COVID-19 vaccine information				<.001				<.001
Media	1440 (26.4)	958 (24.5)	482 (31.0)		1435 (26.4)	922 (24.3)	513 (31.4)	
Nonmedia	4022 (73.6)	2951 (75.5)	1071 (69.0)		4002 (73.6)	2879 (75.7)	1123 (68.6)	
Knowledge of COVID-19 vaccine				<.001				<.001
Level 1	750 (13.7)	476 (12.2)	274 (17.6)		744 (13.7)	458 (12.0)	286 (17.5)	
Level 2	1766 (32.3)	1121 (28.7)	645 (41.5)		1754 (32.3)	1084 (28.5)	670 (41.0)	
Level 3	1237 (22.6)	1018 (26.0)	219 (14.1)		1234 (22.7)	992 (26.1)	242 (14.8)	
Level 4	1709 (31.3)	1294 (33.1)	415 (26.7)		1705 (31.4)	1267 (33.3)	438 (26.8)	
Self-perception								
Perceived severity				<.001				<.001
Level 1	1297 (23.7)	1060 (27.1)	237 (15.3)		1294 (23.8)	1036 (27.3)	258 (15.8)	
Level 2	1311 (24.0)	884 (22.6)	427 (27.5)		1307 (24.0)	845 (22.2)	462 (28.2)	
Level 3	970 (17.8)	632 (16.2)	338 (21.8)		961 (17.7)	605 (15.9)	356 (21.8)	
Level 4	1884 (34.5)	1333 (34.1)	551 (35.5)		1875 (34.5)	1315 (34.6)	560 (34.2)	
Perceived susceptibility				<.001				<.001
Level 1	1009 (18.5)	812 (20.8)	197 (12.7)		1003 (18.4)	787 (20.7)	216 (13.2)	
Level 2	893 (16.3)	613 (15.7)	280 (18.0)		890 (16.4)	589 (15.5)	301 (18.4)	
Level 3	1905 (34.9)	1283 (32.8)	622 (40.1)		1901 (35.0)	1244 (32.7)	657 (40.2)	
Level 4	1655 (30.3)	1201 (30.7)	454 (29.2)		1643 (30.2)	1181 (31.1)	462 (28.2)	
Perceived benefits				<.001				<.001
Level 1	357 (6.5)	184 (4.7)	173 (11.1)		356 (6.5)	172 (4.5)	184 (11.2)	
Level 2	2231 (40.8)	1309 (33.5)	922 (59.4)		2208 (40.6)	1235 (32.5)	973 (59.5)	
Level 3	2874 (52.6)	2416 (61.8)	458 (29.5)		2873 (52.8)	2394 (63.0)	479 (29.3)	
Perceived barriers				<.001				<.001
Level 1	908 (16.6)	855 (21.9)	53 (3.4)		907 (16.7)	846 (22.3)	61 (3.7)	
Level 2	1650 (30.2)	1376 (35.2)	274 (17.6)		1648 (30.3)	1361 (35.8)	287 (17.5)	
Level 3	648 (11.9)	392 (10.0)	256 (16.5)		644 (11.8)	372 (9.8)	272 (16.6)	
Level 4	2256 (41.3)	1286 (32.9)	970 (62.5)		2238 (41.2)	1222 (32.1)	1016 (62.1)	
Self-efficacy				<.001				<.001
Level 1	393 (7.2)	91 (2.3)	302 (19.4)		384 (7.1)	84 (2.2)	300 (18.3)	
Level 2	1795 (32.9)	914 (23.4)	881 (56.7)		1782 (32.8)	855 (22.5)	927 (56.7)	
Level 3	3274 (59.9)	2904 (74.3)	370 (23.8)		3271 (60.2)	2862 (75.3)	409 (25.0)	
Cues to action				<.001				<.001
Multiple channels	2886 (52.8)	2127 (54.4)	759 (48.9)		2864 (52.7)	2064 (54.3)	800 (48.9)	
Policy incentives	61 (1.1)	41 (1.0)	20 (1.3)		62 (1.1)	41 (1.1)	21 (1.3)	
Personal experience	548 (10.0)	297 (7.6)	251 (16.2)		547 (10.1)	272 (7.2)	275 (16.8)	
Social pressure	71 (1.3)	30 (0.8)	41 (2.6)		69 (1.3)	29 (0.8)	40 (2.4)	
No	1896 (34.7)	1414 (36.2)	482 (31.0)		1895 (34.9)	1395 (36.7)	500 (30.6)	
Trust in medical system								
Trust in doctors	34.5 (4.7)	34.9 (4.7)	33.3 (4.4)	<.001	34.5 (4.7)	34.9 (4.7)	33.4 (4.3)	<.001
Trust in vaccine developers	18.4 (3.9)	19.2 (3.8)	16.4 (3.3)	<.001	18.4 (3.9)	19.3 (3.8)	16.4 (3.3)	<.001
Convenience of vaccination								
Distance to the vaccination point (km)				<.001				<.001
<1	2914 (53.4)	2029 (51.9)	885 (57.0)		2904 (53.4)	1957 (51.5)	947 (57.9)	
1‐5	1348 (24.7)	919 (23.5)	429 (27.6)		1347 (24.8)	901 (23.7)	446 (27.3)	
≥5	1200 (22.0)	961 (24.6)	239 (15.4)		1186 (21.8)	943 (24.8)	243 (14.9)	
Time to the vaccination point (min)				.004				.002
<30	5040 (92.3)	3581 (91.6)	1459 (93.9)		5017 (92.3)	3481 (91.6)	1536 (93.9)	
30‐60	383 (7.0)	302 (7.7)	81 (5.2)		381 (7.0)	296 (7.8)	85 (5.2)	
≥60	39 (0.7)	26 (0.7)	13 (0.8)		39 (0.7)	24 (0.6)	15 (0.9)	

a The category boundaries were as follows: vaccine knowledge: level 1: 0‐1, level 2: 2‐3, level 3: 4‐5, and level 4: ≥6; perceived severity: level 1: ≤8, level 2: 9, level 3: 10‐11, and level 4: ≥12; perceived susceptibility: level 1: ≤6, level 2: 7‐8, level 3: 9‐10, and level 4: ≥11; perceived benefits: level 1: ≤8, level 2: 9‐11, and level 3: ≥12; perceived barriers: level 1: ≤7, level 2: 8‐9, level 3: 10‐11, and level 4: ≥12; and self-efficacy: level 1: ≤11, level 2: 12‐15, and level 3: ≥16. Higher levels indicate higher scores. Because of the tied scores, perceived benefits and self-efficacy had 3 observed levels rather than 4.

b Not applicable.

c 1 CNY=US $0.15 as of September 22, 2026.

### Factors Associated With Current and Future CBVH

Table 2 presents the variance components, clustering measures, and model fit statistics for the 4 models. In model 0, the combined household- and community-level ICCs were 77.73% (95% CI 72.40%‐82.29%) for current CBVH and 78.84% (95% CI 73.57%‐83.30%) for future CBVH, supporting the use of 3-level models. After controlling for all variables, the ICCs remained high at 71.94% (95% CI 66.30%‐76.96%) and 72.52% (95% CI 66.97%‐77.45%), respectively. From model 0 to model 3, community-level variance decreased substantially (current CBVH: 3.89-1.36 and future CBVH: 4.29-1.49), whereas household-level variance changed modestly (current CBVH: 7.60-7.07 and future CBVH: 7.97-7.19). In model 3, the MORs for current and future CBVH were 15.96 (95% CI 11.32‐23.62) and 16.62 (95% CI 11.75‐24.70), respectively. This indicates a median 16-fold difference in the odds of hesitancy when comparing 2 individuals with identical covariates from different household-community contexts. Overall, relative to model 0, model 3 accounted for 26.57% and 29.17% of the higher-level variance for current and future CBVH, with most of the reduction occurring at the community level.

**Table 2.** Variance components, clustering measures, and model fit statistics for current and future COVID-19 booster vaccine hesitancy (CBVH).

Model parameters	Model 0	Model 1	Model 2	Model 3
Current CBVH (n=5462)				
Variance components (95% CI)				
Community level (σ^2^_C_)	3.89 (1.98‐7.62)	3.00 (1.47‐6.11)	2.79 (1.36‐5.75)	1.36 (0.59‐3.16)
Household level (σ^2^_H_)	7.60 (6.10‐9.46)	7.08 (5.56‐9.02)	7.05 (5.53‐8.99)	7.07 (5.54‐9.02)
Total (σ^2^_T_)	11.48 (8.63‐15.28)	10.08 (7.46‐13.61)	9.84 (7.31‐13.25)	8.43 (6.47‐10.99)
Variance partitioning, % (95% CI)				
Community level (VPC_C_^a^)	26.30 (15.43‐41.09)	22.41 (12.62‐36.62)	21.27 (11.79‐35.32)	11.64 (5.45‐23.14)
Household level (VPC_H_)	51.43 (41.52‐61.24)	52.98 (43.79‐61.97)	53.67 (44.60‐62.51)	60.30 (52.58‐67.53)
Total (ICC_T_^b^)	77.73 (72.40‐82.29)	75.39 (69.41‐80.54)	74.95 (68.95‐80.11)	71.94 (66.30‐76.96)
Heterogeneity and change				
Total MOR^c^ (95% CI)	25.34 (16.48‐41.65)	20.66 (13.54‐33.76)	19.93 (13.18‐32.22)	15.96 (11.32‐23.62)
Total PCV^d^ (%)	Reference	12.24	14.31	26.57
Model fit				
LL^e^	−2572.24	−1963.02	−1958.84	−1953.53
Deviance, –2LL	5144.49	3926.04	3917.67	3907.06
AIC^f^	5150.49	4016.04	4013.67	4017.06
BIC^g^	5170.30	4313.29	4330.74	4380.37
Future CBVH (n=5437)				
Variance components (95% CI)				
Community level (σ^2^_C_)	4.29 (2.20‐8.36)	3.32 (1.63‐6.74)	3.13 (1.52‐6.42)	1.49 (0.66‐3.39)
Household level (σ^2^_H_)	7.97 (6.40‐9.92)	7.23 (5.69‐9.18)	7.15 (5.62‐9.08)	7.19 (5.65‐9.15)
Total (σ^2^_T_)	12.26 (9.16‐16.41)	10.54 (7.76‐14.31)	10.27 (7.58‐13.92)	8.68 (6.67‐11.30)
Variance partitioning, % (95% CI)				
Community level (VPC_C_)	27.59 (16.43‐42.48)	23.97 (13.64‐38.63)	23.05 (12.94‐37.64)	12.47 (5.98‐24.18)
Household level (VPC_H_)	51.25 (41.19‐61.22)	52.24 (42.77‐61.56)	52.70 (43.31‐61.89)	60.05 (52.23‐67.39)
Total (ICC_T_)	78.84 (73.57‐83.30)	76.21 (70.24‐81.31)	75.74 (69.74‐80.88)	72.52 (66.97‐77.45)
Heterogeneity and change				
Total MOR (95% CI)	28.21 (17.93‐47.67)	22.13 (14.27‐36.90)	21.27 (13.83‐35.11)	16.62 (11.75‐24.70)
Total PCV (%)	Reference	14.01	16.20	29.17
Model fit				
LL	−2617.15	−1995.68	−1992.54	−1986.48
Deviance, –2LL	5234.31	3991.36	3985.08	3972.95
AIC	5240.31	4081.36	4081.08	4082.95
BIC	5260.11	4378.41	4397.92	4446.01

a VPC: variance partition coefficient.

b ICC: intraclass correlation coefficient.

c MOR: median odds ratio.

d PCV: proportional change in variance.

e LL: log-likelihood.

f AIC: Akaike information criterion.

g BIC: Bayesian information criterion.

As shown in Figure 2, in the current CBVH analytical sample, participants with level 3 self-efficacy (aOR 0.01, 95% CI 0.01‐0.02) and level 3 perceived benefits (aOR 0.45, 95% CI 0.25‐0.81) had lower odds of CBVH than those at level 1. Conversely, participants with level 4 perceived barriers (aOR 4.81, 95% CI 2.41‐9.64) and level 4 perceived severity (aOR 2.82, 95% CI 1.74‐4.57) had higher odds than those at level 1. Compared with receiving cues to action from multiple channels, cues derived from social pressure (aOR 2.93, 95% CI 1.08‐7.94) were associated with higher odds of CBVH. Regarding trust in the medical system, higher trust in doctors (aOR 0.97, 95% CI 0.94‐0.99) and in vaccine developers (aOR 0.89, 95% CI 0.84‐0.94) were both associated with lower odds of CBVH. Participants with level 3 vaccine knowledge also had lower odds than those at level 1 (aOR 0.48, 95% CI 0.30‐0.76). Several sociodemographic and health characteristics were also associated with CBVH. Compared with those living with others, individuals living alone had lower odds of CBVH (aOR 0.28, 95% CI 0.10‐0.78). Compared with residents in eastern China, those living in the central (aOR 0.12, 95% CI 0.03‐0.47) and northeastern regions (aOR 0.15, 95% CI 0.03‐0.89) exhibited lower odds of CBVH. Prior COVID-19 vaccination was also associated with lower odds of CBVH (aOR 0.12, 95% CI 0.06‐0.23). Conversely, higher annual household income was associated with increased odds of CBVH (aOR 1.01, 95% CI 1.00‐1.01). Additionally, having chronic diseases (aOR 1.48, 95% CI 1.04‐2.12) or a history of allergies (aOR 1.66, 95% CI 1.04‐2.66) was associated with increased odds of CBVH. Similar associations were observed in the future CBVH analytical sample.

**Figure 2.**
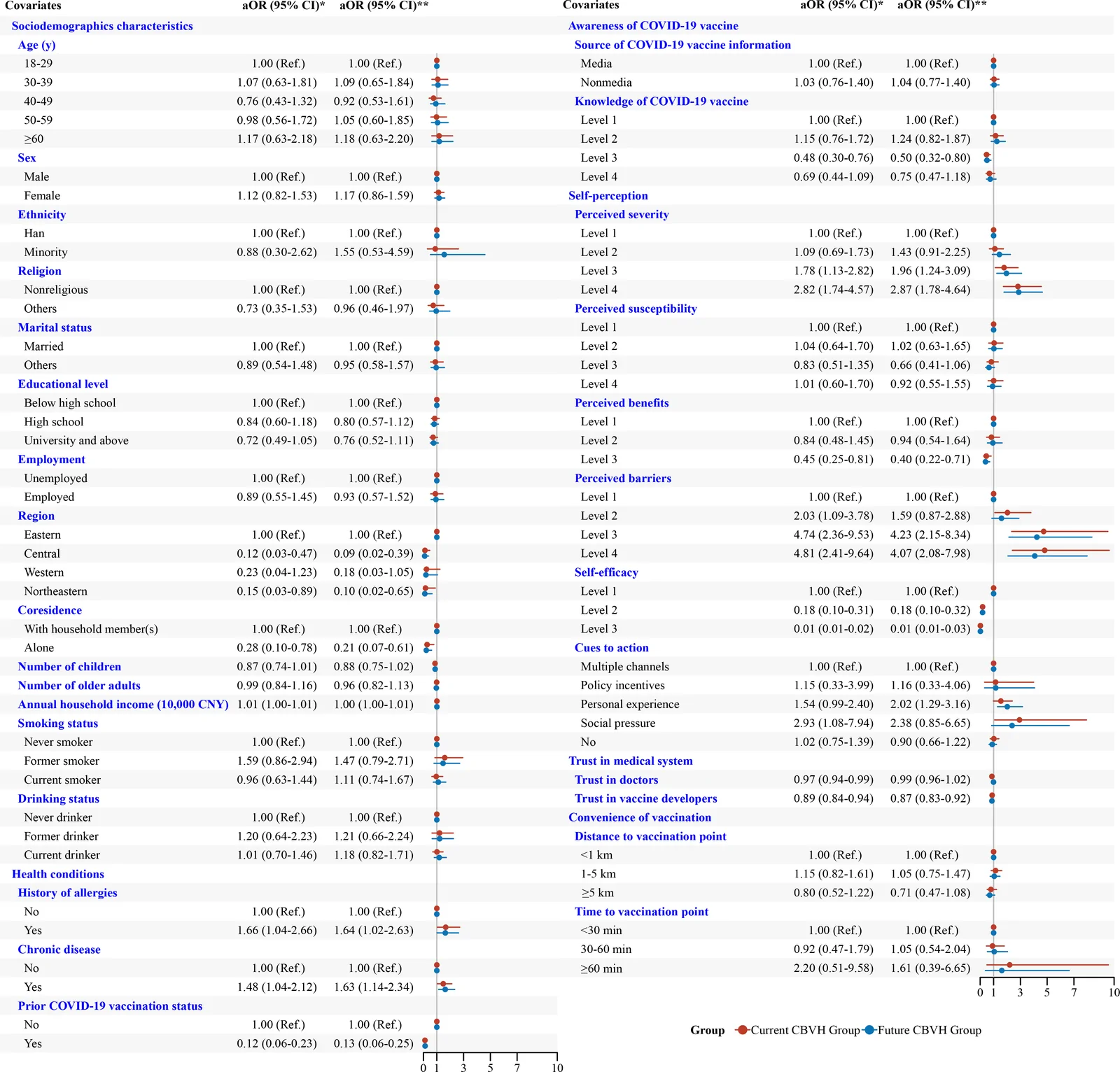
Multilevel logistic regression results for factors associated with current and future COVID-19 booster vaccine hesitancy (CBVH). Adjusted for age, sex, ethnicity, religion, marital status, educational level, employment status, coresidence status, smoking status, drinking status, chronic diseases, history of allergies, prior COVID-19 vaccination status, source of COVID-19 vaccine information, knowledge of the COVID-19 vaccine, perceived severity, perceived susceptibility, perceived benefits, perceived barriers, self-efficacy, cues to action, trust in doctors, trust in vaccine developers, number of children, number of older adults, annual household income, region, distance to the vaccination point, and time to the vaccination point. The category boundaries were as follows: vaccine knowledge: level 1: 0‐1, level 2: 2‐3, level 3: 4‐5, and level 4: ≥6; perceived severity: level 1: ≤8, level 2: 9, level 3: 10‐11, and level 4: ≥12; perceived susceptibility: level 1: ≤6, level 2: 7‐8, level 3: 9‐10, and level 4: ≥11; perceived benefits: level 1: ≤8, level 2: 9‐11, and level 3: ≥12; perceived barriers: level 1: ≤7, level 2: 8‐9, level 3: 10‐11, and level 4: ≥12; and self-efficacy: level 1: ≤11, level 2: 12‐15, and level 3: ≥16. “*” and red lines indicate estimates from the current CBVH multilevel logistic regression model, whereas “**” and blue lines represent estimates from the future CBVH multilevel logistic regression model. aOR: adjusted odds ratio.

### Network Structure and Conditional Associations With CBVH

The network analysis included 14 nodes and is presented in Figure 3. The current and future CBVH networks retained 43 and 41 of the 91 possible edges, corresponding to network densities of 47.3% (43/91) and 45.1% (41/91), respectively. The CC of the CBVH node was 79.6% (4350/5462) in the current network and 79.3% (4311/5437) in the future network. Overall, the 2 networks showed similar configurations.

**Figure 3.**
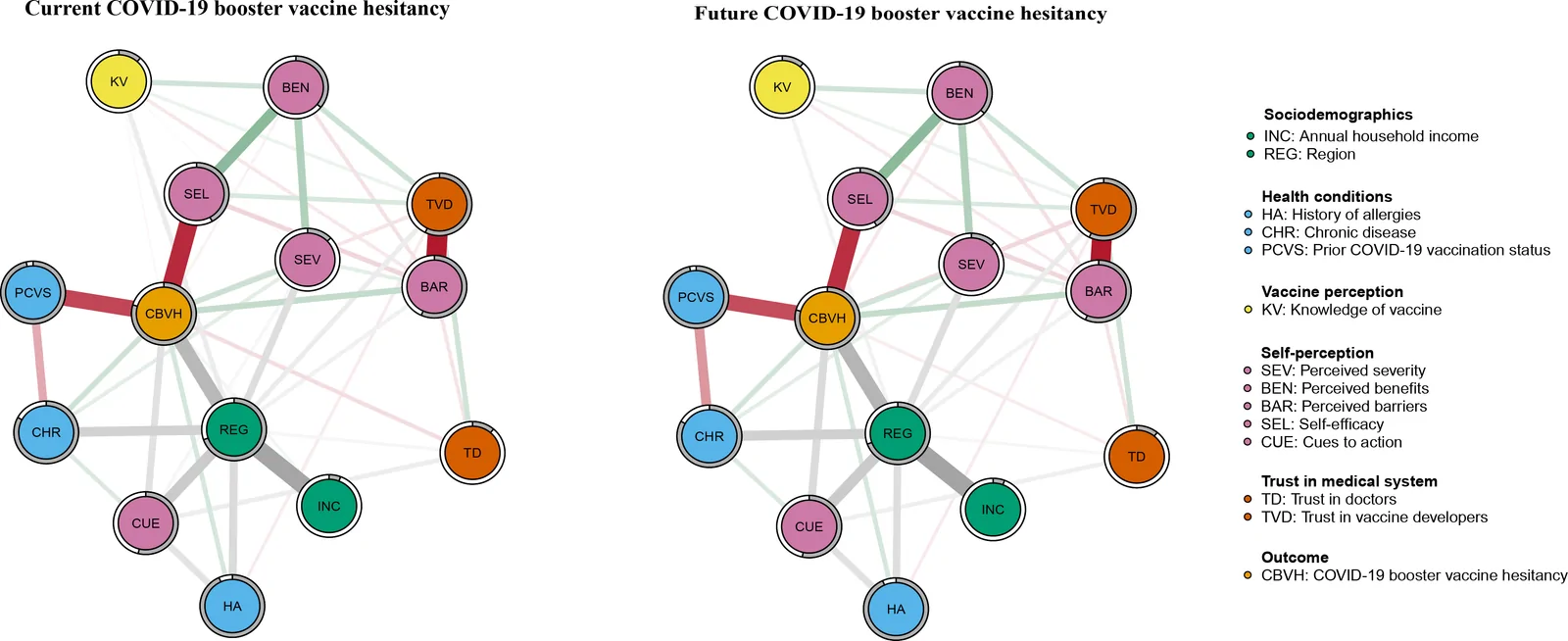
Networks of factors associated with current and future COVID-19 booster vaccine hesitancy. Green and red edges indicate positive and negative conditional associations, respectively, with thicker edges representing larger absolute edge weights. Edges involving multicategorical variables are shown in gray because their category-specific parameters were summarized by the mean of their absolute magnitude and therefore have no single direction. Node colors indicate variable domains. The ring surrounding each node represents its statistical predictability from adjacent nodes, quantified using explained variance (*R^2^*) for continuous variables and classification accuracy for categorical variables.

The magnitudes of conditional associations between study factors and CBVH are presented in Figure 4. In both networks, self-efficacy (current CBVH: −0.548 and future CBVH: −0.533) and prior COVID-19 vaccination status (current CBVH: −0.476 and future CBVH: −0.457) showed the largest conditional associations with CBVH. Region (current CBVH: 0.406 and future CBVH: 0.430) and cues to action (current CBVH: 0.176 and future CBVH: 0.207) also showed large edge weights. Because these are multicategorical variables, the values represent absolute edge magnitudes without a single direction. Edge weights for perceived barriers, perceived severity, chronic disease, and allergy history varied little between networks. Trust in doctors had a larger absolute association with current CBVH (−0.088 vs −0.052), whereas trust in vaccine developers (−0.069 vs −0.099) and perceived benefits (−0.035 vs −0.063) had larger absolute associations with future CBVH. Beyond the CBVH-related edges, the strongest edge weight in both networks was observed between perceived barriers and trust in vaccine developers (weight=0.593).

**Figure 4.**
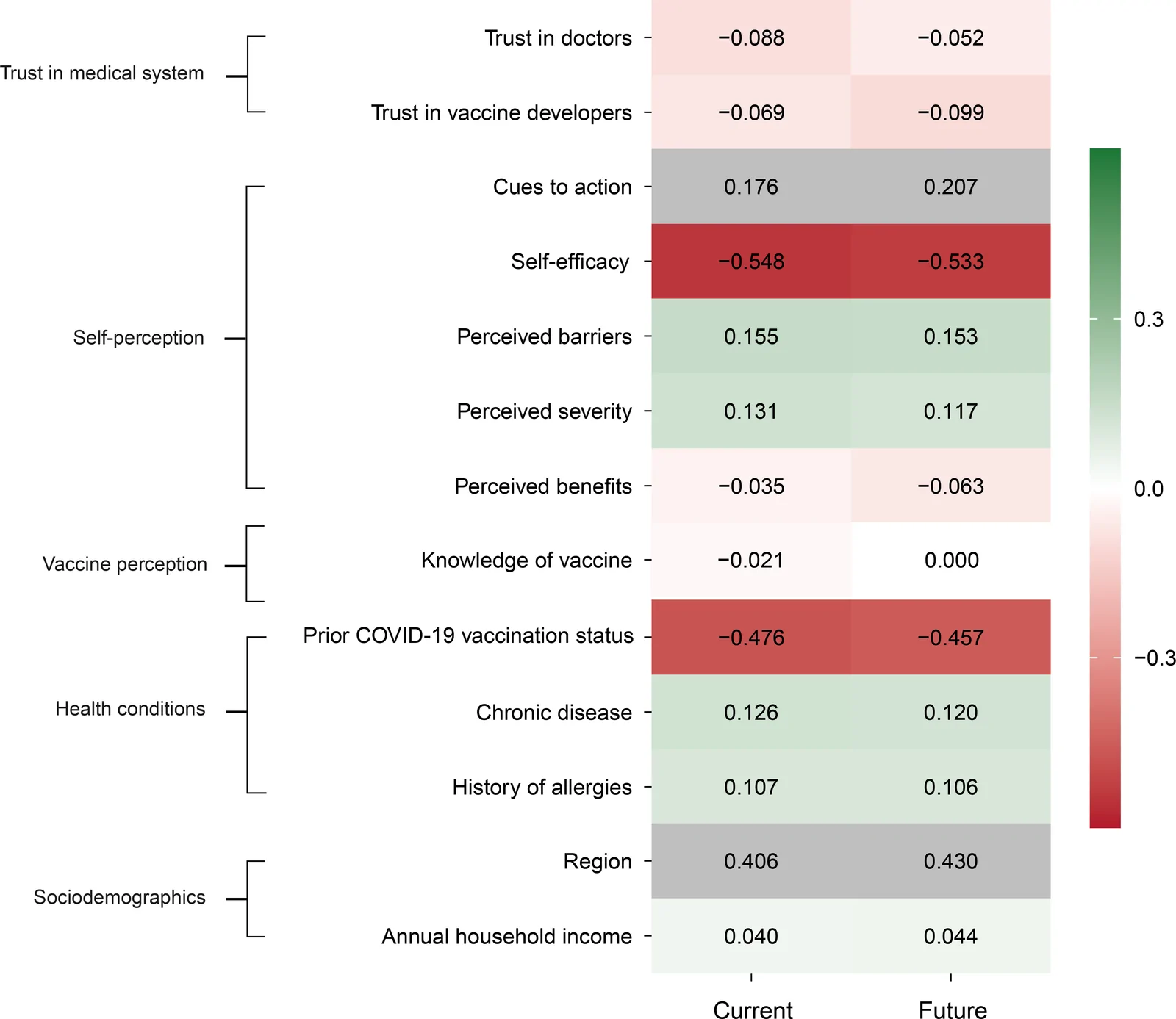
Conditional associations of factors with current and future COVID-19 booster vaccine hesitancy (CBVH). Values represent edge weights for the conditional associations between each study factor and the CBVH node after accounting for all other variables in the mixed graphical model. For continuous and binary variables, positive and negative values indicate the direction of the statistical association, while larger absolute values indicate stronger associations. For the multicategorical variables region and cues to action, values represent the mean absolute magnitude of the category-specific parameters and therefore do not indicate a single direction; these cells are shown in gray. A value of zero indicates that no direct edge with CBVH was retained after regularization.

Centrality indices and bootstrap results are provided in Figures S2 to S4 in Multimedia Appendix 1. The case-dropping bootstrap analysis indicated stability for strength (CS-C: current CBVH=0.750 and future CBVH=0.672) and betweenness (CS-C: current CBVH=0.672 and future CBVH=0.672). Nonparametric bootstrap analysis supported the accuracy of the strongest edge estimates.

### Sensitivity Analysis

Three sensitivity analyses were performed, separately excluding participants with religious beliefs, those with a history of allergies, and those from the eastern region (Tables S2-S4 in Multimedia Appendix 1). The factors associated with CBVH were largely consistent with those in the primary analysis. Additionally, the combined household- and community-level ICCs ranged from 64.96% to 74.15% across the 3 analyses, remaining comparable to the primary estimates of 71.94% for current and 72.52% for future CBVH.

## Discussion

### Principal Findings

This study investigated factors associated with CBVH in a sample from 4 provinces in China, finding that 27.0% (1461/5407) exhibited concurrent CBVH, while the poststratification prevalence of current and future CBVH was 30.88% and 32.55%, respectively. Our findings revealed substantial clustering of CBVH at both the household and community levels (77.73% for current and 78.84% for future). Additionally, network analysis showed that self-efficacy and prior COVID-19 vaccination had the strongest conditional associations with CBVH. The strongest association between non-CBVH nodes was between perceived barriers and trust in vaccine developers. The current and future networks showed broadly similar configurations, supporting the prioritization of factors consistently associated with both outcomes. In general, our results provide insights for developing targeted interventions at the household and community levels to enhance vaccination coverage and strengthen pandemic preparedness.

The observed clustering aligns with social norms theory, which posits that behavior is not only driven by personal motivation or legal enforcement but also influenced by perceptions of others’ behaviors and expectations [32]. At the community level, 2 mechanisms may explain the clustering. First, social selection may produce residential homogeneity. People living in the same community often share similar sociodemographic characteristics, health literacy, and cultural values, which may lead to similar vaccine attitudes [33]. Residents may also face shared structural barriers, including distance to vaccination sites, limited transportation options, unequal access to digital systems, and uneven implementation of public health measures [34,35]. Second, social contagion may spread vaccine-related information and attitudes through neighbors, local groups, and social networks. Vaccine hesitancy may cluster geographically when hesitant views are widespread within a community [36,37]. At the household level, the unique relational structure of the family may make these mechanisms more pronounced. Families often operate under a centralized decision-making hierarchy, whereby one member (eg, parents or adult children) assumes primary responsibility for health decisions [38]. A previous study found that when the family decision-maker was hesitant, 65.1% of other household members tended to follow suit [13]. Notably, household-level variance decreased only modestly after adjustment for measured household characteristics, including household size and income. Future studies should examine other household-level factors, such as decision-making patterns, shared values, and family communication [13,39].

Our findings highlight several key factors associated with CBVH. Higher vaccine knowledge was associated with lower hesitancy, consistent with studies showing that limited vaccine understanding increases hesitancy [40,41]. Self-perception factors, including self-efficacy, perceived benefits, and perceived barriers, were also associated with CBVH [9,42]. Therefore, future interventions may prioritize these modifiable self-perception factors. For instance, motivational interviewing [43] or targeted health education [44] may strengthen vaccination self-efficacy and perceived benefits, while transparent communication regarding vaccine safety and efficacy may help improve perceived benefits [45]. Additionally, we observed a positive association between perceived severity and CBVH. One possible explanation is that during severe outbreaks, individuals may rely more on nonvaccine protective behaviors, such as improved hygiene, reduced mobility, and timely testing, which may be associated with a reduced perceived need for vaccination [46]. Higher trust in doctors and vaccine developers was associated with lower CBVH, underscoring the importance of credible health communication [47]. By contrast, chronic diseases and a history of allergies were associated with higher hesitancy, possibly because these participants were more concerned about their health conditions and adverse reactions [48,49]. Similarly, cues to action were associated with CBVH and have been verified in previous studies [50,51]. Compared with cues from multiple channels, cues based mainly on personal experience or social pressure were linked to higher hesitancy. Living alone was associated with lower CBVH in our study. However, the subgroup of participants living alone was small, and future studies with larger samples are needed to confirm this association. Notably, residents in central and northeastern China, as well as lower-income households, exhibited lower CBVH rates. This may be attributable to the free provision of COVID-19 booster vaccines in China [52], which alleviates financial barriers. This pattern is also consistent with the inverse equity hypothesis, which suggests that hesitancy may emerge first among more socioeconomically advantaged groups [53]. Given the markedly higher CBVH observed in eastern China, we repeated the analysis after excluding this region. Substantial clustering persisted, suggesting that the observed clustering was not driven solely by regional composition.

The network analysis illustrated interrelationships among factors associated with CBVH. Among the factors directly linked to CBVH, self-efficacy showed the strongest association. This finding is consistent with health behavior frameworks in which self-efficacy has been associated with preventive behaviors [54]. Prior COVID-19 vaccination status showed the second strongest direct association. This accords with evidence that vaccination history is associated with booster acceptance and subsequent uptake [55]. Region and cues to action also showed large edge weights. Although these multicategorical estimates did not indicate a uniform direction, they suggest that regional and communication-related factors warrant consideration alongside psychological factors. Beyond the edges directly connected to CBVH, perceived barriers and trust in vaccine developers had the strongest edge in both networks, suggesting that safety and effectiveness may be closely linked to confidence in vaccine producers. A prior study also showed that individuals with less concern about infection severity and lower trust in vaccine effectiveness were more likely to be hesitant [56]. The current and future networks were similar. These findings highlight factors that may warrant further evaluation in intervention studies.

Nevertheless, several limitations should be acknowledged. First, the cross-sectional design limits causal inference, and longitudinal studies are needed to confirm the observed relationships. Second, because CBVH was not a rare outcome, the odds ratios do not approximate prevalence ratios and should be interpreted as measures of association. Third, CBVH was assessed via self-report, which may have introduced reporting bias or social desirability effects. Finally, although our sampling strategy ensured geographic diversity across 4 major provinces, substantial regional heterogeneity may limit the generalizability of the findings.

### Conclusions

In this multicenter household-based study, we found that vaccine hesitancy clustered substantially within households and communities, highlighting the importance of household- and community-level interventions. Self-efficacy and prior vaccination were most directly associated with hesitancy, while perceived barriers and trust in vaccine developers showed the strongest associations within the network. Effective strategies such as regular family-centered vaccine education delivered through community platforms, community-based vaccine counseling services, and transparent dissemination of vaccine-related information and policies may help address vaccine hesitancy. These insights offer a framework for strengthening vaccination strategies and preparedness in future infectious disease outbreaks.

## Supplementary material

10.2196/104263 Multimedia Appendix 1 Measurement items, sensitivity analyses, prevalence estimates, and network analysis results.

10.2196/104263 Checklist 1 STROBE checklist.

## Abbreviations

AIC: Akaike information criterion
aOR: adjusted odds ratio
BIC: Bayesian information criterion
CBVH: COVID-19 booster vaccine hesitancy
CC: proportion of correct classifications
CS-C: correlation stability coefficient
ICC: intraclass correlation coefficient
LL: log-likelihood
MOR: median odds ratio
PCV: proportional change in variance
STROBE: Strengthening the Reporting of Observational Studies in Epidemiology
VPC: variance partition coefficient
