# Supplementary material for: Household and Community Clustering of Concurrent Vaccine Hesitancy in China: Multicenter Survey
Source: JMIR Public Health Surveill. 2026 Oct 7;12:e104263. doi: 10.2196/104263 (PMC13645339; doi:10.2196/104263)
Supplement: Supplementary file 2 [file publichealth-v12-e104263-s001.docx]

***Supplementary Material***

**Household and Community Clustering of Concurrent Vaccine Hesitancy in China: A Multicenter Survey**

[Supplementary Table S1. Items, response format, and internal consistency of the multi-item Health Belief Model and trust constructs.	2](#_Toc28046)

[Supplementary Table S2. Sensitivity analysis of multilevel logistic regression for current and future COVID-19 booster vaccine hesitancy after excluding participants with religious beliefs.	4](#_Toc5108)

[Supplementary Table S3. Sensitivity analysis of multilevel logistic regression for current and future COVID-19 booster vaccine hesitancy after excluding participants with a history of allergies.	8](#_Toc19678)

[Supplementary Table S4. Sensitivity analysis of multilevel logistic regression for current and future COVID-19 booster vaccine hesitancy after excluding participants from the Eastern region.	12](#_Toc18881)

[Supplementary Figure S1. Unweighted and post-stratified prevalence of current and future COVID-19 booster vaccine hesitancy.	16](#_Toc6243)

[Supplementary Figure S2. Centrality measures in the current and future CBVH networks.	17](#_Toc26632)

[Supplementary Figure S3. Stability of centrality indices by case dropping subset bootstrap.	18](#_Toc25638)

[Supplementary Figure S4. Bootstrapped confidence intervals of edge weights.	19](#_Toc5526)

# **Supplementary Table S1. Items, response format, and internal consistency of the multi-item Health Belief Model and trust constructs.**

| **Construct** | **Item wording** | **Response format** | **Cronbach's α** |
| --- | --- | --- | --- |
| Perceived severity | 1. Infection with COVID-19 is fatal.  2. There are many sequelae after COVID-19 is cured.  3. Novel coronavirus infection will greatly affect life. | 5-point Likert scale: 1 = strongly disagree, 2 = disagree, 3 = neutral, 4 = agree, 5 = strongly agree. | 0.891 |
| Perceived susceptibility | 1. The COVID-19 epidemic situation in my area is severe.  2. I have a good chance of contact with the novel coronavirus.  3. I am at high risk of contracting COVID-19. |  | 0.848 |
| Perceived benefits | 1. A booster dose of COVID-19 vaccine can significantly reduce the risk of novel coronavirus infection.  2. Vaccination strengthens the health benefits of those around me.  3. Getting a booster shot of the COVID-19 vaccine will help me get back to my normal life before the pandemic. |  | 0.889 |
| Perceived barriers | 1. I don't trust the manufacturers of the booster shot.  2. I don't trust the vaccinator of the booster shot.  3. I am concerned about the safety of the booster shot.  4. I am concerned about the effectiveness of the COVID-19 booster shot. |  | 0.932 |
| Self-efficacy | 1. Even if it takes time away from work or study, I will get a booster shot.  2. Even if no one around me gets it, I will get a booster shot.  3. Even if I have a fear of needles, I will get it.  4. Even if I still need to take protective measures after vaccination, I will get it. |  | 0.967 |
| Trust in doctors | 1. I trust the medical staff.  2. The medical staff didn't really care about me. (R)  3. The medical staff have my best interests at heart.  4. The medical staff don't respect me. (R)  5. The medical staff wanted to do their best.  6. The medical staff don't know about my life. (R)  7. Medical staff often make mistakes. (R)  8. Medical staff choose this profession for the money. (R)  9. The medical staff would give me the vaccine even if it were bad for me. (R) |  | 0.873 |
| Trust in vaccine developers | 1. Vaccine developers put vaccine safety first.  2. I don't trust the vaccine developers. (R)  3. Vaccine developers just want to make money. (R)  4. Vaccine developers are not interested in helping others. (R)  5. Vaccine developers failed to properly check vaccine safety. (R) |  | 0.925 |

*Notes:* All items were rated on a 5-point Likert scale (1 = strongly disagree to 5 = strongly agree). (R) denotes reverse-scored items; after reverse-scoring, a higher total score indicates a higher level of the construct (for perceived barriers, a higher score indicates greater perceived barriers/lower trust). Each construct score was calculated as the sum of its item scores. Cronbach’s $\alpha$ was calculated in the study sample.

# **Supplementary Table S2. Sensitivity analysis of multilevel logistic regression for current and future COVID-19 booster vaccine hesitancy after excluding participants with religious beliefs.**

| **Covariates** | **Current CBVH aOR (95% CI)** | **Future CBVH aOR (95% CI)** |
| --- | --- | --- |
| **All participants** | **n = 5212** | **n = 5189** |
| **Sociodemographic characteristics** |  |  |
| Age (y) |  |  |
| 18-29 | Ref. | Ref. |
| 30-39 | 1.22 (0.71, 2.09) | 1.18 (0.69, 2.03) |
| 40-49 | 0.85 (0.48, 1.50) | 1.07 (0.60, 1.89) |
| 50-59 | 1.11 (0.62, 1.98) | 1.18 (0.66, 2.10) |
| ≥60 | 1.33 (0.70, 2.52) | 1.43 (0.75, 2.74) |
| Sex |  |  |
| Male | Ref. | Ref. |
| Female | 1.11 (0.81, 1.51) | 1.15 (0.83, 1.57) |
| Ethnicity |  |  |
| Han | Ref. | Ref. |
| Minority | 0.57 (0.15, 2.20) | 1.23 (0.30, 5.01) |
| Marital status |  |  |
| Married | Ref. | Ref. |
| Others | 1.01 (0.60, 1.68) | 1.09 (0.65, 1.84) |
| Educational level |  |  |
| Below high school | Ref. | Ref. |
| High school | 0.87 (0.62, 1.23) | 0.82 (0.58, 1.16) |
| University and above | 0.73 (0.50, 1.08) | 0.80 (0.54, 1.18) |
| Employment |  |  |
| Unemployed | Ref. | Ref. |
| Employed | 0.81 (0.49, 1.34) | 0.89 (0.54, 1.46) |
| Region |  |  |
| Eastern | Ref. | Ref. |
| Central | 0.12 (0.03, 0.48)^**^ | 0.09 (0.02, 0.38)^**^ |
| Western | 0.26 (0.05, 1.41) | 0.22 (0.04, 1.27) |
| Northeastern | 0.15 (0.03, 0.89)^*^ | 0.10 (0.02, 0.65)^*^ |
| Coresidence |  |  |
| With household member(s) | Ref. | Ref. |
| Alone | 0.26 (0.09, 0.73)^*^ | 0.20 (0.07, 0.56)^**^ |
| Number of children | 0.87 (0.74, 1.02) | 0.90 (0.77, 1.06) |
| Number of older adults | 0.97 (0.82, 1.15) | 0.95 (0.80, 1.12) |
| Annual household income (10,000 CNY) | 1.01 (1.00, 1.01) | 1.00 (1.00, 1.01) |
| Smoking status |  |  |
| Never smoker | Ref. | Ref. |
| Former smoker | 1.54 (0.82, 2.87) | 1.49 (0.79, 2.80) |
| Current smoker | 0.92 (0.61, 1.40) | 1.06 (0.70, 1.61) |
| Drinking status |  |  |
| Never drinker | Ref. | Ref. |
| Former drinker | 1.16 (0.62, 2.18) | 1.15 (0.61, 2.16) |
| Current drinker | 1.00 (0.68, 1.46) | 1.13 (0.77, 1.65) |
| **Health conditions** |  |  |
| History of allergies |  |  |
| No | Ref. | Ref. |
| Yes | 1.61 (1.00, 2.61) | 1.61 (0.99, 2.63) |
| Chronic disease |  |  |
| No | Ref. | Ref. |
| Yes | 1.55 (1.08, 2.24)^*^ | 1.64 (1.13, 2.38)^**^ |
| Prior COVID-19 vaccination status |  |  |
| Unvaccinated | Ref. | Ref. |
| Vaccinated | 0.11 (0.05, 0.22)^***^ | 0.12 (0.06, 0.23)^***^ |
| **Awareness of COVID-19 vaccine** |  |  |
| Source of COVID-19 vaccine information |  |  |
| Media | Ref. | Ref. |
| Nonmedia | 1.01 (0.74, 1.36) | 1.01 (0.74, 1.37) |
| Knowledge of COVID-19 vaccine |  |  |
| Level 1 | Ref. | Ref. |
| Level 2 | 1.23 (0.81, 1.86) | 1.24 (0.81, 1.89) |
| Level 3 | 0.49 (0.30, 0.78)^**^ | 0.47 (0.29, 0.75)^**^ |
| Level 4 | 0.77 (0.49, 1.23) | 0.76 (0.47, 1.21) |
| **Self-perception** |  |  |
| Perceived severity |  |  |
| Level 1 | Ref. | Ref. |
| Level 2 | 1.07 (0.67, 1.72) | 1.37 (0.86, 2.18) |
| Level 3 | 1.77 (1.11, 2.83)^*^ | 1.94 (1.21, 3.10)^**^ |
| Level 4 | 2.91 (1.78, 4.76)^***^ | 2.85 (1.74, 4.66)^***^ |
| Perceived susceptibility |  |  |
| Level 1 | Ref. | Ref. |
| Level 2 | 1.00 (0.61, 1.65) | 0.99 (0.60, 1.62) |
| Level 3 | 0.83 (0.50, 1.36) | 0.67 (0.41, 1.09) |
| Level 4 | 1.02 (0.60, 1.74) | 0.94 (0.55, 1.61) |
| Perceived benefits |  |  |
| Level 1 | Ref. | Ref. |
| Level 2 | 0.82 (0.47, 1.43) | 0.91 (0.52, 1.61) |
| Level 3 | 0.45 (0.25, 0.82)^**^ | 0.40 (0.22, 0.73)^**^ |
| Perceived barriers |  |  |
| Level 1 | Ref. | Ref. |
| Level 2 | 2.10 (1.12, 3.92)^*^ | 1.65 (0.90, 3.04) |
| Level 3 | 4.60 (2.27, 9.31)^***^ | 4.04 (2.02, 8.09)^***^ |
| Level 4 | 4.75 (2.35, 9.58)^***^ | 4.01 (2.01, 7.98)^***^ |
| Self-efficacy |  |  |
| Level 1 | Ref. | Ref. |
| Level 2 | 0.19 (0.10, 0.33)^***^ | 0.20 (0.11, 0.35)^***^ |
| Level 3 | 0.01 (0.01, 0.02)^***^ | 0.01 (0.01, 0.03)^***^ |
| Cues to action |  |  |
| Multiple channels | Ref. | Ref. |
| Policy incentives | 1.20 (0.34, 4.25) | 1.24 (0.34, 4.45) |
| Personal experience | 1.63 (1.04, 2.55)^*^ | 2.05 (1.30, 3.23)^**^ |
| Social pressure | 3.20 (1.15, 8.88)^*^ | 2.29 (0.81, 6.52) |
| No | 1.01 (0.74, 1.39) | 0.89 (0.65, 1.22) |
| **Trust in medical system** |  |  |
| Trust in doctors | 0.97 (0.94, 1.00)^*^ | 0.99 (0.96, 1.03) |
| Trust in vaccine developers | 0.89 (0.85, 0.94)^***^ | 0.87 (0.83, 0.92)^***^ |
| **Convenience of vaccination** |  |  |
| Distance to vaccination point |  |  |
| <1 km | Ref. | Ref. |
| 1-5 km | 1.19 (0.84, 1.68) | 1.08 (0.77, 1.53) |
| ≥5 km | 0.83 (0.54, 1.28) | 0.75 (0.48, 1.15) |
| Time to vaccination point |  |  |
| <30 min | Ref. | Ref. |
| 30-60 min | 0.93 (0.47, 1.85) | 0.99 (0.50, 1.98) |
| ≥60 min | 2.34 (0.53, 10.31) | 2.01 (0.46, 8.82) |
| **Random effects** |  |  |
| **Variance components** |  |  |
| Community-level ($\text{σ}_{\text{c}}^{\text{2}}$) | 1.37 (0.59, 3.17) | 1.50 (0.66, 3.42) |
| Household-level ($\text{σ}_{H}^{\text{2}}$) | 6.98 (5.44, 8.96) | 7.35 (5.74, 9.41) |
| Total ($\text{σ}_{T}^{\text{2}}$) | 8.35 (6.37, 10.94) | 8.85 (6.76, 11.58) |
| **Variance partitioning (%)** |  |  |
| Community-level (${VPC}_{C}$) | 11.76 (5.51, 23.35) | 12.39 (5.93, 24.07) |
| Household-level (${VPC}_{H}$) | 59.97 (52.15, 67.30) | 60.51 (52.61, 67.90) |
| Total (${ICC}_{T}$) | 71.73 (65.95, 76.87) | 72.90 (67.28, 77.87) |

*Notes.* Adjusted for age, sex, ethnicity, marital status, educational level, employment status, coresidence status, smoking status, drinking status, chronic diseases, history of allergies, prior COVID-19 vaccination status, source of COVID-19 vaccine information, knowledge of COVID-19 vaccine, perceived severity, perceived susceptibility, perceived benefits, perceived barriers, self-efficacy, cues to action, trust in doctors, trust in vaccine developers, number of children, number of older adults, annual household income, region, distance to vaccination point, and time to vaccination point. The category boundaries were as follows: vaccine knowledge, Level 1 = 0-1, Level 2 = 2-3, Level 3 = 4-5, and Level 4 ≥ 6; perceived severity, Level 1 ≤ 8, Level 2 = 9, Level 3 = 10-11, and Level 4 ≥ 12; perceived susceptibility, Level 1 ≤ 6, Level 2 = 7-8, Level 3 = 9-10, and Level 4 ≥ 11; perceived benefits, Level 1 ≤ 8, Level 2 = 9-11, and Level 3 ≥ 12; perceived barriers, Level 1 ≤ 7, Level 2 = 8-9, Level 3 = 10-11, and Level 4 ≥ 12; and self-efficacy, Level 1 ≤ 11, Level 2 = 12-15, and Level 3 ≥ 16. ^*^*p* < 0.05, ^**^*p* < 0.01, ^***^*p* < 0.001.

# **Supplementary Table S3. Sensitivity analysis of multilevel logistic regression for current and future COVID-19 booster vaccine hesitancy after excluding participants with a history of allergies.**

| **Covariates** | **Current CBVH aOR (95% CI)** | **Future CBVH aOR (95% CI)** |
| --- | --- | --- |
| **All participants** | **n = 5093** | **n = 5067** |
| **Sociodemographic characteristics** |  |  |
| Age (y) |  |  |
| 18-29 | Ref. | Ref. |
| 30-39 | 1.09 (0.61, 1.93) | 1.12 (0.64, 1.94) |
| 40-49 | 0.65 (0.36, 1.20) | 0.86 (0.48, 1.56) |
| 50-59 | 0.94 (0.51, 1.73) | 1.05 (0.58, 1.89) |
| ≥60 | 1.08 (0.55, 2.12) | 1.15 (0.60, 2.23) |
| Sex |  |  |
| Male | Ref. | Ref. |
| Female | 1.08 (0.78, 1.51) | 1.15 (0.83, 1.60) |
| Ethnicity |  |  |
| Han | Ref. | Ref. |
| Minority | 0.70 (0.22, 2.29) | 1.55 (0.49, 4.92) |
| Religion |  |  |
| Nonreligious | Ref. | Ref. |
| Others | 0.77 (0.34, 1.71) | 0.95 (0.43, 2.06) |
| Marital status |  |  |
| Married | Ref. | Ref. |
| Others | 0.94 (0.54, 1.63) | 1.00 (0.59, 1.72) |
| Educational level |  |  |
| Below high school | Ref. | Ref. |
| High school | 0.81 (0.56, 1.16) | 0.78 (0.55, 1.11) |
| University and above | 0.66 (0.44, 0.99)^*^ | 0.72 (0.48, 1.08) |
| Employment |  |  |
| Unemployed | Ref. | Ref. |
| Employed | 0.85 (0.50, 1.46) | 0.92 (0.55, 1.57) |
| Region |  |  |
| Eastern | Ref. | Ref. |
| Central | 0.11 (0.02, 0.49)^**^ | 0.09 (0.02, 0.36)^***^ |
| Western | 0.27 (0.04, 1.65) | 0.21 (0.04, 1.20) |
| Northeastern | 0.14 (0.02, 0.93)^*^ | 0.10 (0.02, 0.62)^*^ |
| Coresidence |  |  |
| With household member(s) | Ref. | Ref. |
| Alone | 0.29 (0.09, 0.88)^*^ | 0.18 (0.06, 0.55)^**^ |
| Number of children | 0.85 (0.71, 1.01) | 0.85 (0.72, 1.01) |
| Number of older adults | 0.97 (0.81, 1.16) | 0.94 (0.79, 1.12) |
| Annual household income (10,000 CNY) | 1.01 (1.00, 1.01)^*^ | 1.00 (1.00, 1.01) |
| Smoking status |  |  |
| Never smoker | Ref. | Ref. |
| Former smoker | 1.78 (0.92, 3.43) | 1.75 (0.92, 3.35) |
| Current smoker | 0.99 (0.64, 1.54) | 1.16 (0.75, 1.78) |
| Drinking status |  |  |
| Never drinker | Ref. | Ref. |
| Former drinker | 1.15 (0.59, 2.22) | 1.07 (0.56, 2.05) |
| Current drinker | 0.97 (0.65, 1.46) | 1.14 (0.77, 1.69) |
| **Health conditions** |  |  |
| Chronic disease |  |  |
| No | Ref. | Ref. |
| Yes | 1.63 (1.10, 2.41)^*^ | 1.72 (1.17, 2.54)^**^ |
| Prior COVID-19 vaccination status |  |  |
| Unvaccinated | Ref. | Ref. |
| Vaccinated | 0.10 (0.05, 0.20)^***^ | 0.11 (0.05, 0.23)^***^ |
| **Awareness of COVID-19 vaccine** |  |  |
| Source of COVID-19 vaccine information |  |  |
| Media | Ref. | Ref. |
| Nonmedia | 0.96 (0.69, 1.33) | 0.96 (0.70, 1.33) |
| Knowledge of COVID-19 vaccine |  |  |
| Level 1 | Ref. | Ref. |
| Level 2 | 1.15 (0.74, 1.78) | 1.35 (0.87, 2.09) |
| Level 3 | 0.41 (0.25, 0.68)^***^ | 0.48 (0.29, 0.78)^**^ |
| Level 4 | 0.68 (0.42, 1.11) | 0.79 (0.49, 1.28) |
| **Self-perception** |  |  |
| Perceived severity |  |  |
| Level 1 | Ref. | Ref. |
| Level 2 | 0.96 (0.58, 1.58) | 1.27 (0.78, 2.06) |
| Level 3 | 1.70 (1.03, 2.81)^*^ | 1.77 (1.09, 2.89)^*^ |
| Level 4 | 2.78 (1.66, 4.68)^***^ | 2.60 (1.56, 4.31)^***^ |
| Perceived susceptibility |  |  |
| Level 1 | Ref. | Ref. |
| Level 2 | 1.06 (0.62, 1.80) | 1.06 (0.64, 1.77) |
| Level 3 | 0.78 (0.46, 1.32) | 0.66 (0.40, 1.10) |
| Level 4 | 0.89 (0.50, 1.58) | 0.90 (0.52, 1.56) |
| Perceived benefits |  |  |
| Level 1 | Ref. | Ref. |
| Level 2 | 0.79 (0.44, 1.43) | 0.82 (0.46, 1.48) |
| Level 3 | 0.45 (0.24, 0.85)^*^ | 0.38 (0.20, 0.70)^**^ |
| Perceived barriers |  |  |
| Level 1 | Ref. | Ref. |
| Level 2 | 2.18 (1.10, 4.31)^*^ | 1.73 (0.91, 3.30) |
| Level 3 | 5.30 (2.46, 11.40)^***^ | 4.80 (2.31, 9.96)^***^ |
| Level 4 | 5.43 (2.52, 11.67)^***^ | 4.46 (2.15, 9.23)^***^ |
| Self-efficacy |  |  |
| Level 1 | Ref. | Ref. |
| Level 2 | 0.19 (0.10, 0.34)^***^ | 0.19 (0.10, 0.35)^***^ |
| Level 3 | 0.01 (0.01, 0.02)^***^ | 0.01 (0.01, 0.03)^***^ |
| Cues to action |  |  |
| Multiple channels | Ref. | Ref. |
| Policy incentives | 1.63 (0.42, 6.32) | 1.06 (0.27, 4.19) |
| Personal experience | 1.76 (1.09, 2.84)^*^ | 2.11 (1.30, 3.40)^**^ |
| Social pressure | 1.77 (0.60, 5.24) | 1.45 (0.48, 4.38) |
| No | 1.02 (0.73, 1.43) | 0.90 (0.65, 1.25) |
| **Trust in medical system** |  |  |
| Trust in doctors | 0.96 (0.93, 1.00)^*^ | 0.99 (0.96, 1.02) |
| Trust in vaccine developers | 0.88 (0.83, 0.93)^***^ | 0.87 (0.82, 0.92)^***^ |
| **Convenience of vaccination** |  |  |
| Distance to vaccination point |  |  |
| <1 km | Ref. | Ref. |
| 1-5 km | 1.06 (0.74, 1.53) | 0.91 (0.64, 1.31) |
| ≥5 km | 0.85 (0.54, 1.34) | 0.72 (0.47, 1.13) |
| Time to vaccination point |  |  |
| <30 min | Ref. | Ref. |
| 30-60 min | 0.93 (0.45, 1.91) | 1.13 (0.56, 2.26) |
| ≥60min | 2.90 (0.58, 14.42) | 2.77 (0.57, 13.57) |
| **Random effects** |  |  |
| **Variance components** |  |  |
| Community-level ($\text{σ}_{\text{c}}^{\text{2}}$) | 1.61 (0.69, 3.74) | 1.45 (0.63, 3.33) |
| Household-level ($\text{σ}_{H}^{\text{2}}$) | 7.83 (6.03, 10.16) | 7.61 (5.90, 9.83) |
| Total ($\text{σ}_{T}^{\text{2}}$) | 9.44 (7.12, 12.51) | 9.06 (6.89, 11.90) |
| **Variance partitioning (%)** |  |  |
| Community-level (${VPC}_{C}$) | 12.64 (5.94, 24.88) | 11.70 (5.51, 23.15) |
| Household-level (${VPC}_{H}$) | 61.51 (53.18, 69.22) | 61.66 (53.73, 69.01) |
| Total (${ICC}_{T}$) | 74.15 (68.39, 79.18) | 73.36 (67.69, 78.35) |

*Notes.* Adjusted for age, sex, ethnicity, religion, marital status, educational level, employment status, coresidence status, smoking status, drinking status, chronic diseases, prior COVID-19 vaccination status, source of COVID-19 vaccine information, knowledge of COVID-19 vaccine, perceived severity, perceived susceptibility, perceived benefits, perceived barriers, self-efficacy, cues to action, trust in doctors, trust in vaccine developers, number of children, number of older adults, annual household income, region, distance to vaccination point, and time to vaccination point. The category boundaries were as follows: vaccine knowledge, Level 1 = 0-1, Level 2 = 2-3, Level 3 = 4-5, and Level 4 ≥ 6; perceived severity, Level 1 ≤ 8, Level 2 = 9, Level 3 = 10-11, and Level 4 ≥ 12; perceived susceptibility, Level 1 ≤ 6, Level 2 = 7-8, Level 3 = 9-10, and Level 4 ≥ 11; perceived benefits, Level 1 ≤ 8, Level 2 = 9-11, and Level 3 ≥ 12; perceived barriers, Level 1 ≤ 7, Level 2 = 8-9, Level 3 = 10-11, and Level 4 ≥ 12; and self-efficacy, Level 1 ≤ 11, Level 2 = 12-15, and Level 3 ≥ 16. ^*^*p* < 0.05, ^**^*p* < 0.01, ^***^*p* < 0.001.

# **Supplementary Table S4. Sensitivity analysis of multilevel logistic regression for current and future COVID-19 booster vaccine hesitancy after excluding participants from the Eastern region.**

| **Covariates** | **Current CBVH aOR (95% CI)** | **Future CBVH aOR (95% CI)** |
| --- | --- | --- |
| **All participants** | **n = 3390** | **n = 3376** |
| **Sociodemographic characteristics** |  |  |
| Age (y) |  |  |
| 18-29 | Ref. | Ref. |
| 30-39 | 0.96 (0.50, 1.84) | 0.88 (0.46, 1.67) |
| 40-49 | 0.55 (0.27, 1.11) | 0.62 (0.31, 1.24) |
| 50-59 | 1.02 (0.51, 2.04) | 0.97 (0.49, 1.93) |
| ≥60 | 0.91 (0.42, 1.97) | 0.79 (0.37, 1.70) |
| Sex |  |  |
| Male | Ref. | Ref. |
| Female | 1.16 (0.78, 1.73) | 1.42 (0.95, 2.13) |
| Ethnicity |  |  |
| Han | Ref. | Ref. |
| Minority | 0.75 (0.25, 2.31) | 1.24 (0.41, 3.74) |
| Religion |  |  |
| Nonreligious | Ref. | Ref. |
| Others | 0.84 (0.34, 2.08) | 1.20 (0.50, 2.90) |
| Marital status |  |  |
| Married | Ref. | Ref. |
| Others | 0.66 (0.35, 1.27) | 0.69 (0.36, 1.32) |
| Educational level |  |  |
| Below high school | Ref. | Ref. |
| High school | 0.85 (0.55, 1.30) | 0.89 (0.58, 1.36) |
| University and above | 0.87 (0.54, 1.41) | 0.97 (0.60, 1.57) |
| Employment |  |  |
| Unemployed | Ref. | Ref. |
| Employed | 0.91 (0.41, 2.01) | 0.85 (0.39, 1.86) |
| Region |  |  |
| Central | Ref. | Ref. |
| Western | 3.12 (0.68, 14.44) | 2.62 (0.55, 12.41) |
| Northeastern | 2.94 (0.56, 15.36) | 2.48 (0.47, 13.24) |
| Coresidence |  |  |
| With household member(s) | Ref. | Ref. |
| Alone | 0.51 (0.17, 1.54) | 0.42 (0.14, 1.27) |
| Number of children | 1.02 (0.86, 1.21) | 1.00 (0.84, 1.19) |
| Number of older adults | 0.94 (0.77, 1.15) | 0.92 (0.75, 1.11) |
| Annual household income (10,000 CNY) | 1.00 (1.00, 1.01) | 1.00 (1.00, 1.01) |
| Smoking status |  |  |
| Never smoker | Ref. | Ref. |
| Former smoker | 1.93 (0.84, 4.43) | 2.20 (0.96, 5.04) |
| Current smoker | 0.95 (0.56, 1.61) | 1.22 (0.72, 2.07) |
| Drinking status |  |  |
| Never drinker | Ref. | Ref. |
| Former drinker | 0.77 (0.33, 1.78) | 0.83 (0.36, 1.91) |
| Current drinker | 1.14 (0.71, 1.83) | 1.38 (0.86, 2.21) |
| **Health conditions** |  |  |
| History of allergies |  |  |
| No | Ref. | Ref. |
| Yes | 1.99 (1.02, 3.88)^*^ | 2.28 (1.16, 4.47)^*^ |
| Chronic disease |  |  |
| No | Ref. | Ref. |
| Yes | 1.41 (0.82, 2.43) | 1.45 (0.84, 2.51) |
| Prior COVID-19 vaccination status |  |  |
| Unvaccinated | Ref. | Ref. |
| Vaccinated | 0.10 (0.04, 0.24)^***^ | 0.11 (0.05, 0.27)^***^ |
| **Awareness of COVID-19 vaccine** |  |  |
| Source of COVID-19 vaccine information |  |  |
| Media | Ref. | Ref. |
| Nonmedia | 1.10 (0.74, 1.64) | 0.96 (0.65, 1.43) |
| Knowledge of COVID-19 vaccine |  |  |
| Level 1 | Ref. | Ref. |
| Level 2 | 1.02 (0.64, 1.62) | 1.30 (0.81, 2.09) |
| Level 3 | 0.38 (0.21, 0.67)^***^ | 0.53 (0.30, 0.93)^*^ |
| Level 4 | 0.41 (0.23, 0.74)^**^ | 0.52 (0.29, 0.93)^*^ |
| **Self-perception** |  |  |
| Perceived severity |  |  |
| Level 1 | Ref. | Ref. |
| Level 2 | 1.23 (0.67, 2.24) | 1.92 (1.06, 3.47)^*^ |
| Level 3 | 1.75 (0.94, 3.27) | 2.28 (1.23, 4.24)^**^ |
| Level 4 | 2.91 (1.53, 5.54)^**^ | 3.96 (2.08, 7.54)^***^ |
| Perceived susceptibility |  |  |
| Level 1 | Ref. | Ref. |
| Level 2 | 1.68 (0.85, 3.31) | 1.66 (0.85, 3.21) |
| Level 3 | 1.24 (0.63, 2.43) | 0.81 (0.42, 1.57) |
| Level 4 | 0.84 (0.40, 1.75) | 0.77 (0.37, 1.59) |
| Perceived benefits |  |  |
| Level 1 | Ref. | Ref. |
| Level 2 | 0.89 (0.44, 1.82) | 0.76 (0.37, 1.54) |
| Level 3 | 0.51 (0.23, 1.09) | 0.27 (0.12, 0.58)^***^ |
| Perceived barriers |  |  |
| Level 1 | Ref. | Ref. |
| Level 2 | 1.71 (0.67, 4.34) | 1.24 (0.51, 3.02) |
| Level 3 | 3.14 (1.13, 8.71)^*^ | 3.18 (1.21, 8.40)^*^ |
| Level 4 | 5.27 (1.99, 13.98)^***^ | 4.68 (1.85, 11.83)^**^ |
| Self-efficacy |  |  |
| Level 1 | Ref. | Ref. |
| Level 2 | 0.17 (0.08, 0.34)^***^ | 0.19 (0.09, 0.40)^***^ |
| Level 3 | 0.02 (0.01, 0.04)^***^ | 0.02 (0.01, 0.05)^***^ |
| Cues to action |  |  |
| Multiple channels | Ref. | Ref. |
| Policy incentives | 1.43 (0.29, 7.11) | 1.16 (0.22, 6.00) |
| Personal experience | 1.31 (0.69, 2.48) | 1.17 (0.61, 2.22) |
| Social pressure | 6.41 (1.34, 30.78)^*^ | 5.33 (1.06, 26.63)^*^ |
| No | 1.01 (0.68, 1.50) | 0.88 (0.59, 1.29) |
| **Trust in medical system** |  |  |
| Trust in doctors | 0.98 (0.94, 1.02) | 1.02 (0.98, 1.05) |
| Trust in vaccine developers | 0.87 (0.81, 0.93)^***^ | 0.87 (0.81, 0.93)^***^ |
| **Convenience of vaccination** |  |  |
| Distance to vaccination point |  |  |
| <1 km | Ref. | Ref. |
| 1-5 km | 1.40 (0.90, 2.19) | 1.29 (0.82, 2.01) |
| ≥5 km | 1.02 (0.58, 1.79) | 0.74 (0.42, 1.28) |
| Time to vaccination point |  |  |
| <30 min | Ref. | Ref. |
| 30-60 min | 0.70 (0.29, 1.70) | 0.72 (0.29, 1.74) |
| ≥60 min | 4.77 (0.79, 28.63) | 3.48 (0.58, 21.01) |
| **Random effects** |  |  |
| **Variance components** |  |  |
| Community-level ($\text{σ}_{\text{c}}^{\text{2}}$) | 0.80 (0.20, 3.10) | 0.84 (0.22, 3.23) |
| Household-level ($\text{σ}_{H}^{\text{2}}$) | 5.30 (3.73, 7.54) | 5.33 (3.79, 7.48) |
| Total ($\text{σ}_{T}^{\text{2}}$) | 6.10 (4.19, 8.87) | 6.17 (4.27, 8.92) |
| **Variance partitioning (%)** |  |  |
| Community-level (${VPC}_{C}$) | 8.48 (2.36, 26.25) | 8.92 (2.53, 26.99) |
| Household-level (${VPC}_{H}$) | 56.48 (46.27, 66.16) | 56.30 (46.26, 65.86) |
| Total (${ICC}_{T}$) | 64.96 (56.03, 72.95) | 65.23 (56.47, 73.06) |

*Notes.* Adjusted for age, sex, ethnicity, religion, marital status, educational level, employment status, coresidence status, smoking status, drinking status, chronic diseases, history of allergies, prior COVID-19 vaccination status, source of COVID-19 vaccine information, knowledge of COVID-19 vaccine, perceived severity, perceived susceptibility, perceived benefits, perceived barriers, self-efficacy, cues to action, trust in doctors, trust in vaccine developers, number of children, number of older adults, annual household income, region, distance to vaccination point, and time to vaccination point. The category boundaries were as follows: vaccine knowledge, Level 1 = 0-1, Level 2 = 2-3, Level 3 = 4-5, and Level 4 ≥ 6; perceived severity, Level 1 ≤ 8, Level 2 = 9, Level 3 = 10-11, and Level 4 ≥ 12; perceived susceptibility, Level 1 ≤ 6, Level 2 = 7-8, Level 3 = 9-10, and Level 4 ≥ 11; perceived benefits, Level 1 ≤ 8, Level 2 = 9-11, and Level 3 ≥ 12; perceived barriers, Level 1 ≤ 7, Level 2 = 8-9, Level 3 = 10-11, and Level 4 ≥ 12; and self-efficacy, Level 1 ≤ 11, Level 2 = 12-15, and Level 3 ≥ 16. ^*^*p* < 0.05, ^**^*p* < 0.01, ^***^*p* < 0.001.

# Supplementary Figure S1. Unweighted and post-stratified prevalence of current and future COVID-19 booster vaccine hesitancy.

COVID-19 Booster Vaccine HesitancyPrevalence (%)

|  | Unweighted prevalence | Post-stratified prevalence |
| --- | --- | --- |
| Current | 28.43% | 30.88% |
| Future | 30.09% | 32.55% |

*Notes.* Post-stratified prevalence estimates were calculated using weights based on the joint distributions of sampled region, age group, and sex from the Seventh National Population Census of China (2020).

# Supplementary Figure S2. Centrality measures in the current and future CBVH networks.

*Notes.* Centrality indices are presented as standardized z scores, with higher values indicating greater relative centrality. Negative z scores indicate below-average centrality rather than negative associations. Blue and red lines represent the current and future CBVH networks, respectively. Differences between the two networks are descriptive. CBVH, COVID-19 booster vaccine hesitancy; HA, history of allergies; CHR, chronic disease; PCVS, prior COVID-19 vaccination status; INC, annual household income; REG, region; KV, knowledge of COVID-19 vaccine; SEV, perceived severity; BEN, perceived benefits; BAR, perceived barriers; SEL, self-efficacy; CUE, cues to action; TVD, trust in vaccine developers; TD, trust in doctors.

# Supplementary Figure S3. Stability of centrality indices by case dropping subset bootstrap.

Supplementary Figure S3a. Stability of centrality indices within the current CBVH group.

Supplementary Figure S3b. Stability of centrality indices within the future CBVH group.

*Notes.* The x-axis represents the percentage of cases of original sample used at each step. The y-axis represents the average of correlations between the centrality indices from the original network and the centrality indices from the networks that were reestimated after dropping increasing percentages of cases. Each line indicates the correlations of betweenness, closeness, and strength, while areas indicate 95% CI.

# Supplementary Figure S4. Bootstrapped confidence intervals of edge weights.

Supplementary Figure S4a. Bootstrapped confidence intervals of edge weights within the current CBVH group.

Supplementary Figure S4b. Bootstrapped confidence intervals of edge weights within the future CBVH group.

*Notes.* Red dots indicate the sample edge-weight estimates, black dots indicate bootstrap means, and gray areas represent 95% bootstrap CIs. Wider intervals indicate lower estimation precision.
